# Psychological benefits of bilateral and unilateral plyometric training configurations in post-pubertal highly trained male soccer players

**DOI:** 10.3389/fpsyg.2026.1844921

**Published:** 2026-05-11

**Authors:** Achraf Hammami, Raouf Hammami, Abdelkader Mahmoudi, Haithem Rebai, Roland van den Tillaar

**Affiliations:** 1 Higher Institute of Sport and Physical Education of Ksar-Said, University of Manouba, University Campus, Manouba, Tunisia; 2 Tunisian Research Laboratory ‘Sports Performance Optimization', National Center of Medicine and Science in Sports (CNMSS), Tunis, Tunisia; 3 Higher Institute of Sport and Physical Education of Sfax, University of Sfax, University Campus, Sfax, Tunisia; 4 Department of Sport Sciences and Physical Education, Nord University, Levanger, Norway

**Keywords:** autoregulation, football, jump training, mental well-being, training prescription

## Abstract

This study examined the effects of different plyometric training configurations on mental well-being in post-pubertal highly trained male soccer players. Participants were assigned to bilateral (4 × 8; *n* = 10), high-volume unilateral (4 × 8; *n* = 10), or low-volume unilateral (4 × 4; *n* = 11) training groups. Pre-post assessments included cognitive anxiety, somatic anxiety, self-confidence, attention, and emotional competence. Baseline scores were similar across groups for most variables (*p* > 0.05), except total emotional competence, which was higher in the unilateral 4 × 4 group (*F* = 13.4, *p* < 0.001, η^2^ = 0.48). Significant main effects of time were observed for all variables (*F* ≥ 18.7, *p* ≤ 0.001, η^2^ ≥ 0.37). Cognitive anxiety decreased significantly in all groups (*p* < 0.05), while somatic anxiety decreased significantly only in the bilateral group (*p* < 0.05). Self-confidence increased significantly in the bilateral and unilateral 4 × 4 groups (*p* < 0.05). Attention showed a significant time × group interaction (*F* = 3.54, *p* = 0.042, η^2^ = 0.20), with the unilateral 4 × 4 group improving more than the unilateral 4 × 8 group. Emotional competence increased in all groups; however, the unilateral 4 × 4 group remained higher at both pre- and post-test, resulting in a significant group effect (*F* = 16.4, *p* < 0.001, η^2^ = 0.53). No significant differences were found between high- and low-volume unilateral training for the other outcomes. These results indicate that plyometric training enhances mental well-being in youth highly trained male soccer players, but neither increased volume nor unilateral specificity provides additional benefits. Bilateral training may be most effective for reducing cognitive anxiety, while low-volume unilateral training offers a time-efficient alternative.

## 1 Introduction

The psychological development of youth athletes has become an increasingly important focus in sport science, particularly during the post-pubertal stage, when performance demands and psychosocial pressures intensify (Naughton et al., 2000; Guo et al., 2025). In soccer, young players are frequently exposed to competitive stressors that can elevate cognitive and somatic anxiety while requiring high levels of emotional regulation, attentional control, and interpersonal competence (Tassi et al., 2023; Shi et al., 2024; Sánchez-Ruiz et al., 2025). Key constructs such as emotional intelligence and self-confidence have been identified as key determinants of performance and long-term athlete development, influencing decision-making, coping strategies, and social interactions on and off the field (Guo et al., 2025; Yue et al., 2025). Therefore, identifying training strategies that enhance both physical performance and psychological well-being is a priority for coaches and practitioners.

Plyometric training defined as exercises involving rapid stretch—shortening cycle actions, in which an eccentric muscle action is immediately followed by a concentric contraction within a short ground-contact time (Komi and Nicol, 2010) particularly when performed at higher intensities or with greater coordinative and cognitive demands, has been shown to positively affect mental health outcomes in youth soccer players (Znazen et al., 2022; Hammami et al., 2025a). These exercises may improve attentional focus, cognitive engagement, and emotional regulation. Mechanistically, benefits are thought to involve neurobiological adaptations, including modulation of neurotransmitters such as serotonin and dopamine, and increased brain-derived neurotrophic factor, supporting cognitive function and emotional control (Colucci-D'amato et al., 2020). While unilateral and bilateral configurations and differing training volumes have been proposed to differentially affect psychological outcomes, evidence remains limited and inconsistent (Clemente et al., 2025; Hammami et al., 2025a).

Recent evidence suggests that improvements in cognitive anxiety, self-confidence, attention, and emotional competence can occur across various plyometric configurations, even at relatively low volumes, highlighting a potential threshold effect whereby a sufficient stimulus elicits meaningful psychological benefits without requiring maximal training load (Znazen et al., 2022; Hammami et al., 2025a). Lower-volume unilateral protocols may be particularly effective for attentional engagement, possibly due to reduced fatigue and increased focus during exercises with coordinative and cognitive demands. However, baseline variability, sample size, and intervention duration can influence observed effects, emphasizing the need for controlled studies to clarify these relationships.

The present study addresses these gaps by directly comparing the effects of bilateral plyometric training (4 × 8), high-volume unilateral training (4 × 8), and lower-volume unilateral training (4 × 4) on cognitive and somatic anxiety, self-confidence, attention, and emotional intelligence in post-pubertal highly trained male soccer players. By systematically manipulating both laterality and volume within a controlled design, this study investigates whether training configuration influences psychological outcomes beyond general high-intensity exercise benefits. Based on previous research and theoretical frameworks (Znazen et al., 2022; Clemente et al., 2025; Hammami et al., 2025a), it was hypothesized that: (i) all training programs would improve psychological outcomes, (ii) high-volume unilateral training would produce greater improvements in attention and cognitive anxiety due to increased coordinative demands, and (iii) lower-volume unilateral training would elicit comparable, though slightly smaller, benefits, offering a time-efficient approach to enhance mental well-being.

## 2 Methods

### 2.1 Study design

**Figure 1:**
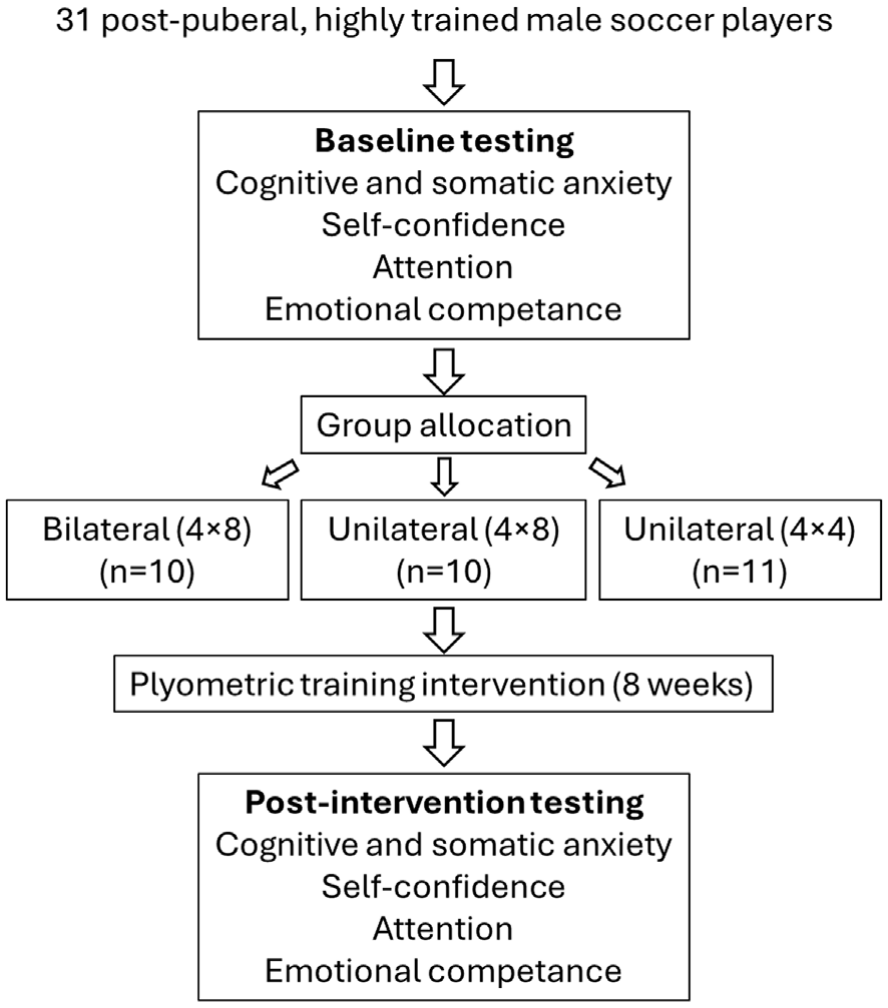
Study design and experimental procedure.

A randomized parallel-group experimental design was used to examine the effects of three bilateral (4 × 8), high (4 × 8) and low volume (4 × 4) unilateral plyometric training programs on cognitive and somatic anxiety, self-confidence, and emotional intelligence (emotional attention, total score, and intra- and interpersonal competence) in post-pubertal highly trained male soccer players. Participants were randomly assigned to one of the three groups. The study was conducted over an intervention period integrated within the regular in-season training schedule. Pre- and post-plyometric training assessments were conducted to measure changes in all psychological parameters, using validated questionnaires to quantify anxiety, self-confidence, and emotional intelligence before and after the training intervention (Figure 1). All post-intervention assessments were administered at least 48–72 h after the final training session, under resting conditions and prior to any exercise, in order to minimize the potential influence of acute fatigue on the results. Statistical analyses included repeated-measures ANOVA to assess main effects of time, group, and time × group interactions, with effect sizes calculated to determine the magnitude of observed changes.

### 2.2 Participants

The sample size estimation was computed using G^*^Power software (version 3.1.6). Based on findings from a related study, (Hammami et al., 2025b) examining the effects of plyometric training on somatic anxiety (Cohen's *f* = 0.44) in highly-trained male adolescent soccer players (Tier 3), an *a priori* power analysis with a type I error of 0.01 and 90% statistical power was computed. Analysis indicated that 32 participants would represent a sufficient sample. All participants were engaged in regular competitive soccer training and had several years of training experience. Inclusion criteria required players to be free from injury in the 3 months preceding the study and to have no history of neurological or psychological disorders. For the 6–8 years preceding the study, the participating young soccer players regularly engaged in general plyometric and conditioning training 2–3 times per week. According to the training and performance caliber framework of Mckay et al. (2021), the study population can be categorized as Tier 3 (highly trained).

Players were randomly assigned to a bilateral (*n* = 10), high (*n* = 10) and low volume (*n* = 11) unilateral plyometric training group. Throughout the study period, all participants continued the same soccer-specific training program under the supervision of the same coaching staff. As such, all participants completed an 8-week progressive training program consisting of two 30-min sessions per week, integrated in their regular soccer training sessions within their total schedule of 5 weekly sessions. Before study participation, the participating young athletes and their parents or legal representatives received a letter containing information about potential benefits and risks of study participation. Parents or legal representatives and athletes signed the consent form after thorough explanation of the objectives and the scope of the study, the procedures, risks, and benefits. This study was conducted following the latest version of the Declaration of Helsinki and the protocol was approved by the local ethics committee of the National Center of Medicine and Science of Sports, Tunis (CNMSS-LR09SEP01) before the commencement of the study. None of the participating athletes had a history of psychological and/or musculoskeletal, neurological, or orthopedic disorders 6 months prior to the start of the study.

### 2.3 Procedures

The study followed a pre-post design, with all variables assessed before (pre-test) and after (post-test) the 8-weeks intervention during in-season period. Baseline testing was conducted under standardized conditions, and participants were familiarized with all procedures prior to data collection. The intervention consisted of plyometric training sessions performed in addition to regular soccer practice. Training frequency, intensity, and total duration were controlled across groups, with the primary difference being the mode of execution (bilateral vs. unilateral) and repetition scheme (4 × 8 vs. 4 × 4). The mental well-being, emotional intelligence, and attention assessments were then conducted after ensuring adequate recovery time.

For the cognitive, somatic anxiety, self-confidence, attention and emotional intelligence assessments, only one trial was performed. All attention and emotional intelligence tests were administered individually in quiet, controlled rooms under the supervision of trained researchers, to ensure standardized conditions and minimize potential distractions. The researchers administering the assessments were not blinded to group allocation, which is acknowledged as a potential source of expectation bias, particularly during the administration of the *d*2 attention test. A passive control group was not included in this study because it is unethical to not allow young athletes to train for a certain period of time (Hammami et al., 2025a,b). Since authors from previous studies have already shown that plyometric training is generally effective for mental well-being enhancement in young soccer players (Hammami et al., 2025a), our main goal was to directly compare the specific effects of different configurations of bilateral versus unilateral plyometric training.

## 3 Anthropometrics and body composition

Athletes' body height and mass were collected by a trained specialist using a wall-mounted stadiometer (Florham Park, NJ) and an electronic scale (Baty International, West Sussex, England), respectively.

The sum of skinfolds was assessed using Harpenden's skinfold calipers. Anthropometric testing was conducted according to Deurenberg et al. (1991) who reported similar prediction errors between adults and adolescents. Thereafter, biological maturity was evaluated non-invasively using chronological age, standing and sitting height as input parameters for a regression equation to subsequently predict the maturity offset (Moore et al., 2015). The following prediction equations were applied: MO = −7.999994 + (0.0036124 × age × height). The equation has previously been validated for boys and presents a standard error of estimate reported as 0.542 years (Moore et al., 2015).

### 3.1 Mental well-being assessment

#### 3.1.1 Tests for the assessment of anxiety and self-confidence

Participants' competitive state anxiety was tested using the Competitive State Anxiety Inventory-2 (CSAI-2). The Arabic translation of the questionnaire, validated with 13 items by Boudhiba et al. (2015) was applied. The CSAI-2 is a widely recognized tool for assessing multi-dimensional anxiety in athletes within competitive environments. This inventory evaluates three core components. First, cognitive anxiety which reflects worries and negative thoughts about performance (e.g., “I am concerned about this competition,” “I am concerned about choking under pressure”); second, somatic anxiety that pertains to the physical symptoms of anxiety, such as increased heart rate or muscle tension (e.g., “I feel nervous,” “I feel tense in my stomach”); and third, self-confidence which represents an athlete's belief in his ability to perform successfully (e.g., “I feel at ease,” “I am confident I can meet the challenge”).

Participants responded to each item on a four-point Likert scale, indicating “how do you feel right now,” with options ranging from 1 (“not at all”) to 4 (“very much so”). Each of the three subscales cognitive anxiety, somatic anxiety, and self-confidence consists of 13 items, resulting in a possible summed score range of 13–52 for each subscale. Scores were calculated by summing the responses for the items within each subscale to provide an intensity level for that component. No percentage transformation was applied; all values represent raw sum scores. This method provides a clear, quantitative measure of each psychological construct, enabling comparisons across groups and over time. Using this approach, researchers can examine how anxiety and self-confidence relate to physical performance and tailor interventions aimed at reducing anxiety and enhancing self-confidence in young athletes. The translated CSAI-2 included 13 items, and it has previously demonstrated excellent test-retest reliability in youth athletes with ICC values of 0.94 for cognitive anxiety, 0.87 for somatic anxiety, and 0.79 for self-confidence, respectively (Reid and Debeliso, 2019).

#### 3.1.2 Tests for the assessment of emotional intelligence

Emotional competence was assessed using the Psychometric Emotional Competence (PEC) scale. Participants responded to 50 items on a five-point Likert scale (1 = strongly disagree, 2 = disagree, 3 = neutral, 4 = agree, 5 = strongly agree), resulting in a possible total score range of 50–250. Higher scores indicate greater emotional competence. For analysis and presentation purposes, the total scores were divided into four categories representing relative levels of emotional competence: low (50–112), moderate (113–175), high (176–225), and very high (226–250) and not percentages, ensuring that the data accurately reflect participants' responses. The Psychometric Emotional Competence measures both intrapersonal emotional competence (understanding one's own emotions) and interpersonal emotional competence (understanding others' emotions) as separate constructs. The instrument also provides a global score representing overall emotional competence. The Psychometric Emotional Competence has previously demonstrated excellent reliability with ICC values ranging between 0.90 and 0.98 for all of the studied items (Aouani et al., 2019).

#### 3.1.3 Tests for the assessment of attention

The d2 test was used to evaluate participants' selective attention, concentration, and mental speed, and is widely recognized for its reliability and validity. The test demonstrates excellent reliability, with ICCs ranging from 0.95 to 0.98 across variables (Aouani et al., 2019), and strong criterion, construct, and predictive validity (Boudhiba et al., 2015). The test consists of 14 lines, each containing 47 letters, including the target letters “*p*” and “*d*” with 1–4 small marks. Participants were instructed to quickly scan each line and cross out every “*d*” with exactly two marks, while ignoring all other letters and symbols. Each line was completed within 20 s. The dependent variable was the total d2 test score, calculated as the number of correctly identified target letters minus the number of errors, reflecting attention and concentration performance.

#### 3.1.4 Plyometric training programs

The lower-body plyometric training intervention was implemented as an 8-week, sport-integrated in-season programs. The jump training program is detailed in Table 1. In line with established definitions of plyometric exercise—i.e., rapid stretch–shortening cycle actions characterized by a fast eccentric phase immediately followed by a concentric contraction with minimal ground-contact (coupling) time—the training interventions were designed to ensure that all jumps met plyometric criteria rather than representing general jumping drills. Hence, participants were instructed to perform each jump with maximal effort, emphasizing rapid ground contact time, correct landing mechanics, and full extension during the concentric phase. They were also encouraged to maintain proper posture, ensure controlled knee alignment during landing, and minimize excessive rest between repetitions while maintaining technical quality. Verbal encouragement and corrective feedback were provided by the supervising coaches to ensure adherence to proper technique and consistent execution across participants. Following the approach of Bogdanis et al. (2019), the bilateral group performed all exercises using both legs simultaneously. To equate training volume with the bilateral condition, the unilateral group performed half the repetitions per leg; total training volume was operationalized as the total number of ground contacts across both limbs per session, rather than simply repetitions. This method was chosen to provide a comparable mechanical stimulus, as each jump represents a discrete load on the neuromuscular system. Session duration, exercise intensity, and rest intervals were matched between groups to ensure comparable training exposure. Both the unilateral and bilateral groups completed two training sessions per week, each lasting approximately 30 min (Table 1).

**Table 1:** Bilateral and unilateral plyometric training programs.

Exercise	Sets	Bilateral reps.	Unilateral reps. per leg	Total foot contacts (bilateral)	Total foot contacts (unilateral)	Rest (between sets)
20 cm drop jump	3	6	3	18	18	1 min
Horizontal jumps	3	10	5	30	30	1 min
Lateral hops	3	10	5	30	30	1 min

Total foot contacts were calculated as sets × reps × number of legs, ensuring that unilateral and bilateral groups received equivalent mechanical loading per session. Repetitions per leg in the unilateral group were halved to match the total ground contacts of the bilateral condition. This operationalization of volume was used to provide a comparable neuromuscular stimulus between groups.

## 4 Statistical analysis

To check for normality, Shapiro–Wilk test of normality was used and confirmed. To assess the effects of using plyometrics upon mental well-being, a two (time:pre-post test, repeated measures) × 3 (groups: bilateral, unilateral 4 × 4 and 4 × 8) analysis of variance (ANOVA) was used. *Post-hoc* testing was conducted using Holm–Bonferroni adjustment for multiple pairwise comparisons following significant omnibus effects. The correction was applied only to *post-hoc* tests, whereas the omnibus ANOVA tests for the five dependent variables were not adjusted. Effect size was evaluated with (Eta partial squared) where 0.01 < η^2^ < 0.06 constitutes a small effect, a medium effect when 0.06 < η^2^ < 0.14 and a large effect when η^2^ > 0.14 (Cohen, 1988). The level of significance was set at *p* ≤ 0.05. Statistical analysis was performed using JASP (version 0.95.1, University of Amsterdam, Amsterdam, Netherlands).

## 5 Results

**Figure 2:**
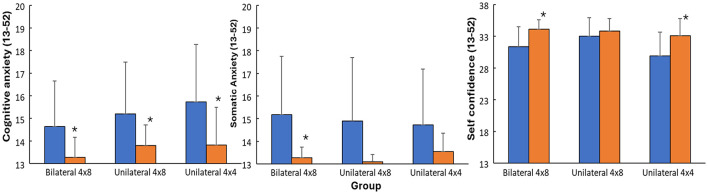
Mean (SD) self-confidence, cognitive and somatic anxiety scores per group at pre and post test. * indicates a significant change from pre- to post test for this group.

No significant difference for the parameters was found between the groups at baseline (*p* > 0.05) except for total emotional competence (*F* = 13.4, *p* < 0.001, η^2^ = 0.48), which was significantly higher in the unilateral 4 × 4 group, compared with the other groups). Significant effect of time (*F* ≥ 18.7, *p* ≤ 0.001, η^2^ ≥ 0.37) was found for all tested parameters, while only a significant group effect on total emotional competence (*F* = 16.4, *p* < 0.001, η^2^ = 0.53), and a significant time × group interaction effect (*F* = 3.54, *p* = 0.042, η^2^ = 0.20) was observed for attention. *Post hoc* comparison revealed that significant decreases in cognitive anxiety in all groups, for somatic anxiety only significant decrease in the bilateral group from pre- to post, while self-confidence increased significantly for in the bilateral and unilateral 4 × 4 groups (Figure 2).

**Figure 3:**
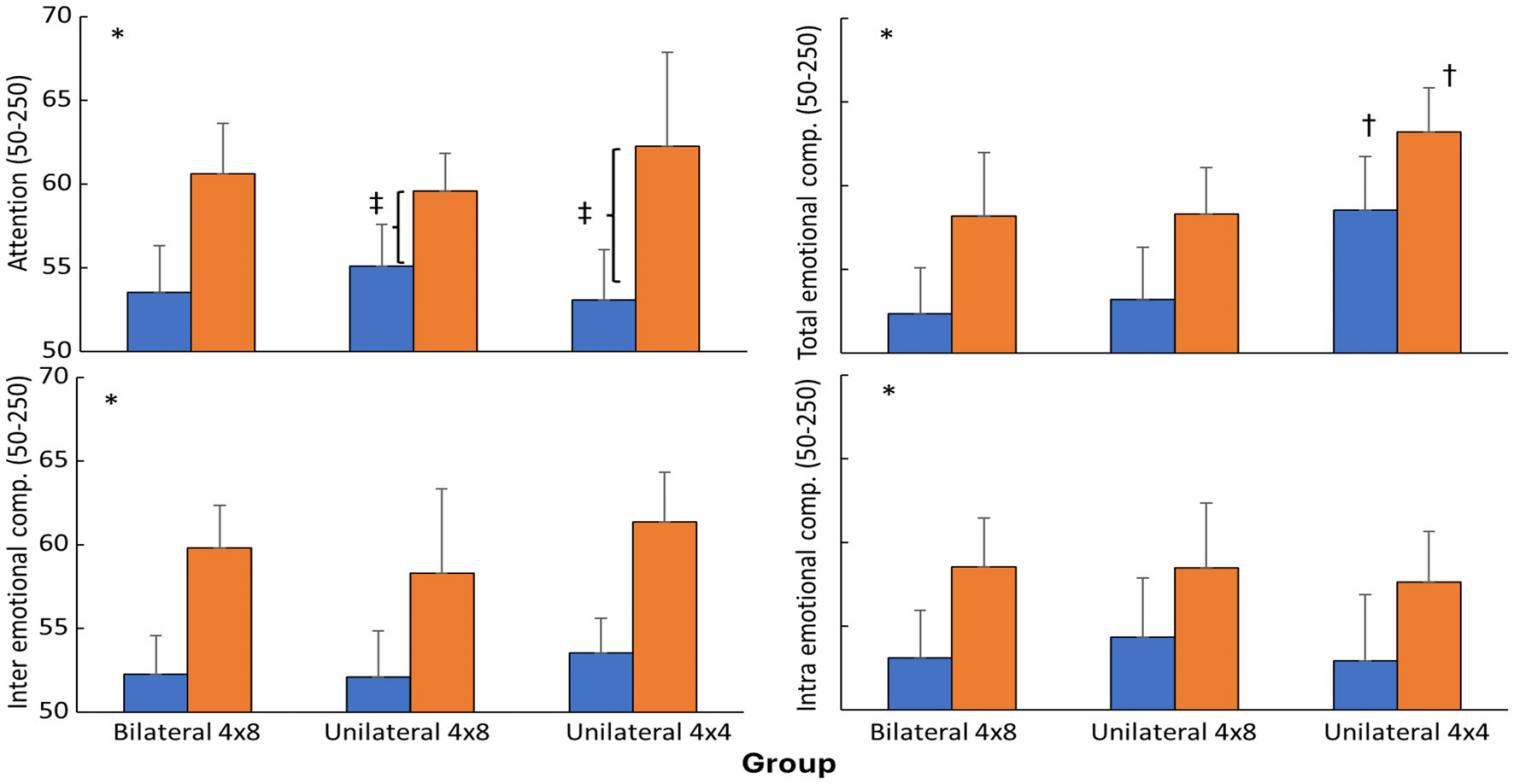
Mean (SD) in emotional intelligence parameters per group at pre and post-test. * indicates a significant change from pre- to post-test for all groups. † indicates a significant higher value for this group at this test with the other two groups. ‡ Indicates a significant difference in increase between these two groups from pre- to post-test.

For the emotional competence variables all groups increased significantly from pre- to post-test. However, total emotional competence for the unilateral 4 × 4 group was significantly higher at pre and post test compared with the other groups, resulting in a group effect. However, the increase from pre- to post-test for this variable was similar between groups, while attention increased significantly higher for the unilateral 4 × 4 group compared with the unilateral 4 × 8 group resulting in a significant interaction effect (Figure 3).

## 6 Discussion

The present study investigated the psychological effects of bilateral plyometric training (4 × 8), high-volume unilateral plyometric training (4 × 8), and lower-volume unilateral plyometric training (4 × 4) in post-pubertal highly trained male soccer players. Across all groups, significant improvements were observed over time in cognitive anxiety, self-confidence, attention, and emotional competence, consistent with evidence that high-intensity exercise enhances psychological outcomes via neurobiological and psychosocial mechanisms, including increased catecholamines, endorphin release, and mastery experiences that improve emotional regulation and self-efficacy (Biddle and Asare, 2011; Lubans et al., 2016).

A notable observation is that baseline scores for several psychological measures, particularly anxiety, were already relatively low, limiting the potential for large reductions due to a possible floor effect. Despite this, cognitive anxiety decreased significantly in all groups, while somatic anxiety decreased significantly only in the bilateral training group. Self-confidence increased significantly in the bilateral and unilateral 4 × 4 groups but not in the unilateral 4 × 8 group, suggesting differential within-group responses.

For emotional competence, all groups improved from pre- to post-test; however, the unilateral 4 × 4 group had significantly higher total emotional competence at both pre- and post-test compared with the other groups, resulting in a significant group effect. Importantly, the magnitude from pre- to post-test changes was similar across groups, indicating that the group effect primarily reflects pre-existing baseline differences rather than differential training-induced gains. This underscores the importance of considering baseline variability when interpreting group comparisons, particularly in small samples where initial differences can disproportionately influence statistical outcomes (Sun et al., 2010).

Attention results were particularly noteworthy, as a significant time × group interaction indicated that the unilateral 4 × 4 group improved more than the unilateral 4 × 8 group. This finding is especially relevant given the initial hypothesis that higher-volume unilateral training would elicit superior attentional benefits due to greater coordinative and cognitive demands. Contrary to expectations, the low-volume unilateral condition produced the most pronounced improvement. One plausible explanation is that higher training volume may have introduced greater neuromuscular and cognitive fatigue, thereby attenuating the quality of attentional engagement during sessions. In contrast, the lower-volume condition may have preserved attentional resources, allowing participants to maintain higher focus, movement quality, and cognitive engagement during execution. Although direct physiological or perceptual load indicators (e.g., RPE or external/internal load markers) were not collected to confirm this mechanism, this interpretation is consistent with evidence that excessive acute load can impair executive function and cognitive performance in youth athletes.

Additionally, it is possible that the lower-volume condition optimized the balance between cognitive challenge and recoverability, thereby producing a more favorable stimulus for attentional adaptation. In this sense, attentional benefits may depend not solely on coordinative complexity, but on the interaction between complexity and fatigue state. Once fatigue exceeds a certain threshold, the quality of cognitive engagement may diminish, limiting the expected psychological gains. This interpretation aligns with emerging perspectives suggesting that “optimal challenge” rather than maximal load is most conducive to cognitive and psychological adaptation in exercise contexts.

Nonetheless, the absence of consistent superiority across other psychological outcomes suggests that neither training volume nor laterality alone systematically determines improvements in mental well-being. Overall, the findings indicate that plyometric training-induced psychological benefits appear largely similar across the tested conditions, within the limits of the present sample and design.

These results may be tentatively interpreted within a threshold-effect framework, whereby a minimum effective stimulus could be sufficient to elicit psychological benefits, and increasing training volume beyond this level may not necessarily produce additional gains. However, this interpretation should be considered exploratory, as evidence for a threshold effect in psychological outcomes remains limited and indirect, and the present study did not directly test dose-response relationships. Further research specifically designed to examine dose-response effects on psychological variables is needed to substantiate this hypothesis.

Potential neurophysiological mechanisms that may underlie these observed effects could include upregulation of brain-derived neurotrophic factor (BDNF), enhanced synaptic plasticity, and possible engagement of prefrontal networks involved in emotional regulation and attentional control (Thiele and Bellgrove, 2018; Caponnetto et al., 2021). These explanations are presented as theoretical interpretations, as no neurobiological biomarkers were measured in the present study. Finally, it is important to acknowledge that the relatively small sample size and baseline variability likely reduced statistical power across outcomes, increasing the risk of Type II error and limiting the detection of subtle between-group differences, despite moderate to large effect sizes (η^2^ up to 0.53). Although *a priori* power analysis was based on somatic anxiety from a previous study, this may not fully represent the required sample size for the other psychological variables (e.g., cognitive anxiety, self-confidence, emotional competence, and attention), which may differ in expected effect magnitudes and variability. Therefore, the study may be underpowered to detect smaller or domain-specific effects across these outcomes.

Accordingly, null or non-significant findings for individual variables should be interpreted with caution, as they may reflect limited statistical power rather than the absence of true effects. Future studies with larger samples and outcome-specific power estimations are needed to more precisely determine the sensitivity of each psychological construct to plyometric training interventions.

Furthermore, the present finding suggests that lower-volume unilateral plyometric training (4 × 4) may be sufficient to elicit meaningful psychological adaptations, including improvements in attention and self-confidence, without requiring higher-volume protocols, particularly within the context of an in-season training environment. In the present study, the intervention was implemented during the competitive (in-season) period, when athletes are concurrently exposed to regular training and match demands. This context should be considered when interpreting the findings, as training timing within the season may influence cognitive and psychological responses. Nevertheless, the observed outcomes suggest that a time-efficient approach may be feasible for supporting mental well-being, allowing coaches to optimize training efficiency, recovery, and overall load management without compromising psychological benefits. Several limitations should be acknowledged. First, the small sample size and baseline group differences limit statistical inference and generalizability. In addition, the sample consisted exclusively of male youth soccer players, which limits the generalizability of the findings to female athletes and mixed-gender populations. Future research should include female and mixed samples to determine whether the observed psychological responses to plyometric training are consistent across sexes. Second, while the study did not include an active control group (e.g., participants engaged only in regular soccer training), this decision can be positively interpreted within the ethical context of youth sports, where ensuring all athletes benefit from structured training is a priority. The observed improvements in cognitive anxiety, emotional competence, and attention are therefore encouraging, as they reflect meaningful development within a real-world training environment. Although it is difficult to attribute these changes exclusively to the plyometric intervention since seasonal training effects and natural maturation may also contribute (Malina et al., 2004) the findings still provide valuable preliminary evidence supporting the potential benefits of integrating such training into youth soccer programs, while highlighting an important direction for future controlled research. Third, the intervention duration may have been insufficient for differential adaptations to emerge, particularly for complex constructs such as emotional competence. Fourth, self-report measures are subject to potential bias from social desirability and subjective interpretation. Finally, findings are specific to post-pubertal male soccer players and may not generalize to other age groups, sexes, or sports (Eime et al., 2013). Finally, similar improvements in attentional focus, cognitive engagement, and emotional regulation could also result from other forms of exercise involving coordinative challenge, novelty, or high physical demand, future research should include comparative intervention designs (e.g., plyometric vs. other coordinatively demanding or resistance-based training) to clarify whether plyometrics confer distinct psychological benefits beyond general effects of structured physical training.

Future research should address these limitations by including larger and more heterogeneous samples, longer intervention periods, and non-training control groups. Combining subjective assessments with objective physiological markers such as cortisol, heart rate variability, and brain-derived neurotrophic factor would deepen understanding of mechanisms underlying psychological adaptations (Gomez-Pinilla and Hillman, 2013). Additionally, examining cognitive load, task complexity, and multimodal training approaches may clarify whether specific configurations uniquely influence mental well-being. Longitudinal designs assessing sustainability and performance relevance of psychological improvements are also recommended.

## 7 Conclusions

The present study demonstrates that plyometric training can positively influence selected aspects of mental well-being, such as cognitive anxiety, self-confidence, attention, and emotional competence, in post-pubertal highly trained male soccer players. Improvements were observed across all groups over time; however, these effects were largely independent of training volume or laterality. Notably, the unilateral 4 × 4 group had higher total emotional competence at both pre- and post-test, creating a group effect, but the magnitude of change was similar across groups, indicating that baseline differences rather than the training itself drove this effect.

Contrary to the initial hypothesis, increasing training volume or emphasizing unilateral execution did not confer superior psychological benefits. Time-dependent improvements occurred regardless of training configuration, suggesting that a minimum effective stimulus is sufficient to elicit meaningful mental well-being adaptations, and that higher volume or unilateral specificity offers no additional advantage within the tested range.

From a practical standpoint, coaches and practitioners can select plyometric training modalities based primarily on physical performance goals, logistical considerations, or fatigue management, without concern for differential psychological outcomes. Lower-volume unilateral protocols appear as effective as higher-volume or bilateral approaches for enhancing mental well-being, offering a time-efficient and flexible training option.

Overall, these findings refine current assumptions by demonstrating that plyometric training modality and volume do not differentially affect most psychological outcomes in youth athletes. Future research should investigate the mechanisms underlying modality-specific responses, examine longer-term adaptations, and explore whether combining plyometric training with cognitive or neuromuscular interventions produces more robust or sustained psychological benefits.

## Data Availability

The raw data supporting the conclusions of this article will be made available by the authors, without undue reservation.
