# Supplementary material for: Psychological benefits of bilateral and unilateral plyometric training configurations in post-pubertal highly trained male soccer players
Source: Front Psychol. 2026 May 11;17:1844921. doi: 10.3389/fpsyg.2026.1844921 (PMC13200614; doi:10.3389/fpsyg.2026.1844921)
Supplement: Supplementary file 1 [file Table_1.DOCX]

**Table.** Mean (95% CI) Anthropometry and psychological variable at pre and post test per group

| **Group** | **Bilateral 4x8** | | **Unilateral 4x8** | | **Unilateral 4x4** | |
| --- | --- | --- | --- | --- | --- | --- |
| time | Pre | Post | Pre | Post | Pre | Post |
| Age (yrs.) | 16.5 (16.3-16.6) | | 16.4 (16.3-16.5) | | 16.6 (16.3-16.9) | |
| Height (m) | 1.69 (1.64-1.72) | | 1.72 (1.68-1.75) | | 1.71 (1.66-1.76) | |
| Body mass (kg) | 62.9 (56.8-69.0) | | 62.5 (58.3-66.6) | | 64.4 (58.8-70.1) | |
| Cognitive anxiety (13-52) | 14.6 (13.4-15.9) | 13.3 (12.7-13.8) | 15.2 (13.8-16.6) | 13.8 (13.2-14.4) | 15.7 (14.2-17.3) | 13.8 (12.8-14.8) |
| Somatic anxiety (13-52) | 15.2 (13.6-16.8) | 13.3 (13.0-13.6) | 14.9(13.2-16.6) | 13.1 (13.0-13.3) | 14.7 (13.2-16.2) | 13.6 (13.0-14.1) |
| Self-confidence (13-52) | 31.4 (29.4-33.3) | 34.1 (33.2-35.0) | 33.0 (31.2-34.8) | 33.8 (32.6-35.0) | 29.9 (27.6-32.2) | 33.1 (31.4-34.7) |
| Attention (50-250) | 53.6 (51.8-55.3) | 60.6 (58.8-62.5) | 55.1 (53.5-56.7) | 59.6(58.2-61.0) | 53.1 (51.2-55.0) | 62.3 (58.8-65.8) |
| Inter-emotional comp. (50-250) | 52.3 (50.8-53.7) | 59.8 (58.2-61.4) | 52.1 (50.4-53.3) | 58.3 (55.2-61.4) | 53.6 (52.3-54.8) | 61.3 (59.5-63.2) |
| Intra-emotional comp. (50-250) | 53.1 (51.3-54.9) | 58.6 (56.7-60.4) | 54.3 (52.1-56.5) | 58.5 (56.1-60.9) | 52.9 (50.5-55.4) | 57.6 (55.8-59.5) |
| Total emotional comp. (50-250) | 52.4 (50.7-54.1) | 58.2 (55.8-60.5) | 53.2 (51.3-55.1) | 58.3 (56.6-60.0) | 58.6 (56.6-60.5) | 63.2 (61.5-64.8) |
